# The patient experience of Colchicine Resistant-Familial Mediterranean Fever (cr-FMF)

**DOI:** 10.1186/1546-0096-13-S1-P22

**Published:** 2015-09-28

**Authors:** P Dandekar, J Gregson, R Campbell, F Bourhis

**Affiliations:** 1 Novartis Pharma AG, Basel, Switzerland; 2 MAPI, Uxbridge, UK; 3 MAPI, Nanterre, France

## Introduction

Familial Mediterranean Fever (FMF) is a genetic disorder characterized by recurrent attacks of fever and pain, which is most common in those of Sephardic Jewish, Armenian, Turkish or Arabic descent. Although colchicine is the mainstay of treatment for FMF, an estimated 5 to 15% of patients have an incomplete response to colchicine (e.g. colchicine-resistance FMF [cr-FMF]).[^1^,^2^]

## Objectives

To determine the impact of cr-FMF on patients’ or caregivers’ lives, to describe patient's journey from first onset of symptoms to present, and to identify areas for improvement in cr-FMF patient care.

## Patients and methods

Patients with cr-FMF or their caregivers (paediatric patients) (N=16) were recruited through rare disease experts and patient support groups. Patients completed a 20 page pre-interview questionnaire and an in-depth 90 minute interview. Complete data (n=14) were quantified with topics focused on symptoms, diagnosis process, treatment experience, treatment needs and impact on wellbeing.

## Results

The majority of patients were adults (n=14) with a family history of FMF (n=14). The disease generally starts in childhood, with 65% of patients experiencing symptoms before 10 years. Attacks occurred with variable frequency, ranged from weekly to every 3-4 months, lasting between 12-72 hours. Reported triggers included physical or emotional stress, or menstruation or occured spontaneously. Commonly reported symptoms were stomach, fever, joint pain, difficulty breathing and chest and back pain. Diagnosis delays were also variable (4 months-44 yrs), with half of patients experiencing delays of ~5 years. Flares were extremely debilitating and patients were often bedridden, leading to missed work and school. Entire families were impacted, especially caregivers. Patients/caregivers were often dependant on family for support and financial assistance. Most patients continue to be treated with colchicine despite only partial response and distressing side-effects. They expressed the need for treatments that provided not only rapid relief during flares, but also prevented or reduced attacks.

## Conclusion

Patients with cr-FMF reported a significant impact of the disease on physical, social, emotional, and practical/financial aspects of their lives. In their journey with cr-FMF, they commonly experienced diagnostic delays and misdiagnoses. Therapeutic options with improved efficacy and fewer side-effects are needed for the treatment of cr-FMF.
